# Bio-inspired nanocomposite coatings on orthodontic archwires with corrosion resistant and antibacterial properties

**DOI:** 10.3389/fbioe.2023.1272527

**Published:** 2023-10-20

**Authors:** Yuming Chen, Zihan Chen, Zebin Zheng, Yong Xia

**Affiliations:** The First Affiliated Hospital of Shantou University Medical College, Shantou, China

**Keywords:** polydopamine-graphene oxide, NiTi alloy, corrosion resistance, antibacterial properties, orthodontic archwire

## Abstract

The corrosion resistance and antibacterial properties of fixed orthodontic devices are insufficient in the complex oral cavity, which delays tooth movement and causes enamel demineralization. To overcome the challenges, this research constructs a series of polydopamine-graphene oxide (PDA-GO) nanocoatings on representative NiTi archwires via self-assembly. The morphology, chemical structure, and multifunctional properties of coatings showed tunability dependent on the PDA/GO ratio. Optimized PDA-GO coatings with uniform and dense characteristics prolonged the diffusion path for the corrosive medium and reduced Ni dissolution in NiTi alloys. Meanwhile, the applied coatings endowed NiTi alloys with antibacterial activity against *Streptococcus mutans* due to the surface structures and inherent properties of PDA-GO. *In vitro* cytotoxicity tests further verified their good biocompatibility. This bio-inspired nanocomposite coating provides a practical reference for modification of dental metal surfaces to better behave in the intraoral environment.

## 1 Introduction

Equiatomic nickel-titanium (NiTi) alloys are frequently employed in orthodontic treatment to align and level dentition. Orally placed for more than 3 months, NiTi archwires continuously interact with saliva proteins, corrosive ions, bending stress, and microorganisms (Ghazal et al., 2015; Wang et al., 2023; Zhang et al., 2023). However, they are ineffective in preventing corrosion and lack antimicrobial qualities, leading to reduced service efficiency and several clinical complications. For example, orthodontic stress intensifies the corrosion behavior of NiTi wires in saliva, further increases the friction coefficient, and prolongs treatment process (Krishnan et al., 2012). Health issues, such as cytotoxicity and allergic responses, may result from excessive Ni^2+^ release into oral cavity (Močnik et al., 2017). Additionally, orthodontic fixtures hamper oral hygiene, combined with adhered bacteria, which poses a risk of enamel demineralization and periodontal diseases (Venkatesan et al., 2020). In this regard, materials science-oriented investigations should pay more attention to the concerns and modifications of dental metals and alloys.

The past couple of decades have witnessed the booming development of nanomaterials in metallic surface design, which could meet the requirements of diverse oral treatment (Moses and Mandal, 2022). Notably, graphene oxide (GO) is suitable for orthodontic mechanical scenario because of its excellent strength and elastic deformation (Yang et al., 2022). The large surface area and special layered structure give it unique advantages in filling pores and improving corrosion resistance of the coating (Zhou et al., 2022). GO can also endow metal surfaces with antibacterial activity through membrane damage and oxidative stress (Lee et al., 2018; Mazinani et al., 2021). However, the bonding durability on metal surface of GO remains a challenge (Han et al., 2018). Some natural compounds have been used to modify substrates to enhance long-term stability, such as mussel-inspired polydopamine (PDA) (Cheng et al., 2019). Combining biomimetic PDA with GO was reported to achieve Ti alloys with tribological properties for orthopaedic applications (Wang et al., 2019); however, the feasibility of these nanosystems as orthodontic coatings in oral environment requires emphatic investigation.

**FIGURE 1:**
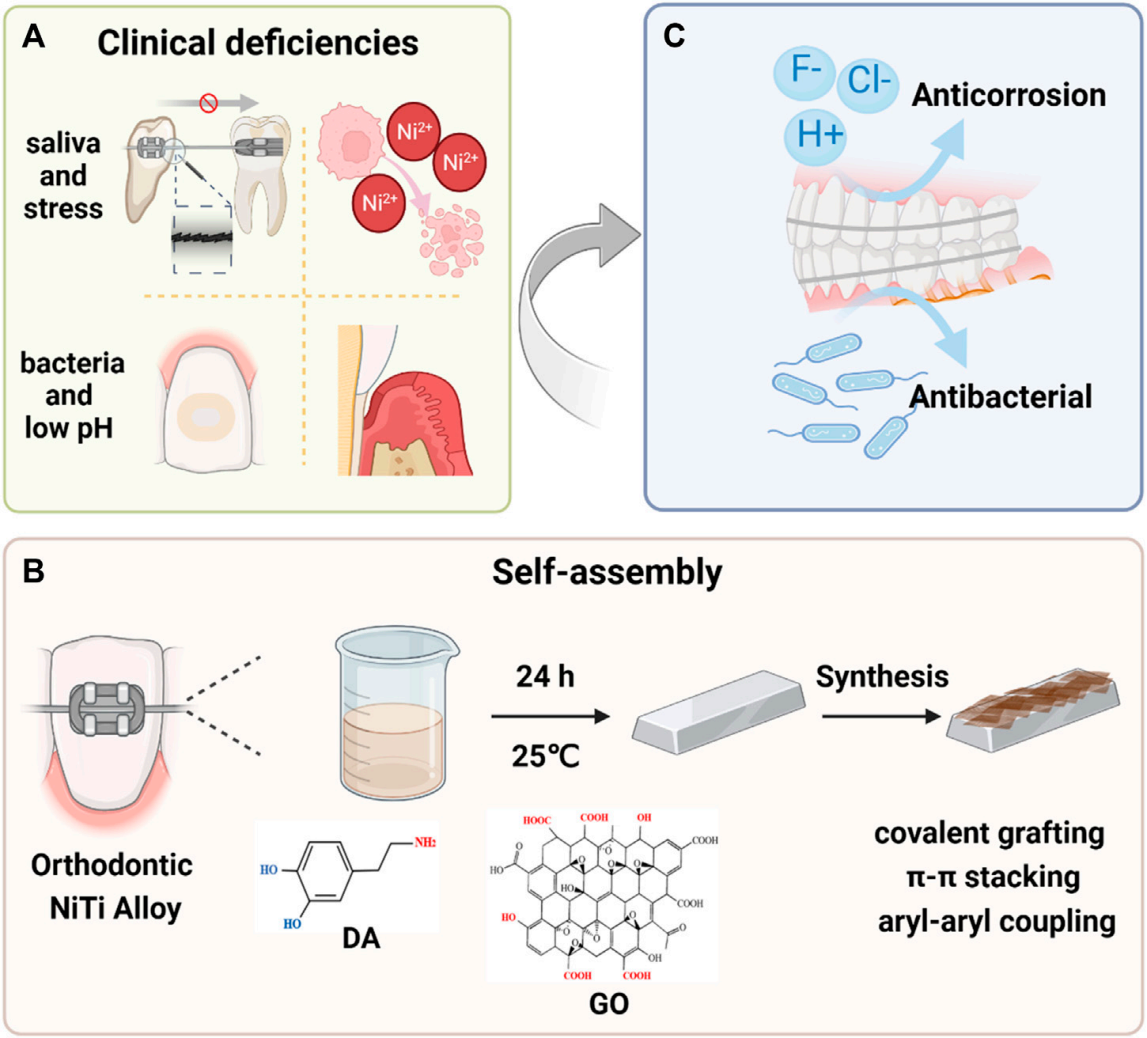
Schematic illustration of the PDA-GO modified orthodontic NiTi archwires. **(A)** Metallic fixed appliances can induce specific changes in oral environment. Stress corrosion under salivary corrosion medium impacts tooth movement and releases excess Ni^2+^. Also, a decreased pH promotes development of bacteria-induced enamel demineralization and periodontal diseases. The most commonly used NiTi alloys have poor corrosion resistance and no antibacterial behavior, which requires modification. **(B)** The modification process and mechanisms of forming PDA-GO coatings from precursors in a self-assembly reaction. **(C)** PDA-GO coatings encourage excellent anti-corrosion performance while preventing bacterial infection.

Self-assembly is an environmentally benign strategy for manufacturing multi-component coatings while preserving the substrates’ mechanical properties (Sun et al., 2023; Tanaka et al., 2023). It provides a powerful method to exploit desired functionality based on intermolecular interactions (Lee et al., 2019). Herein, the PDA-GO coatings were conducted on orthodontic NiTi alloys by self-assembly technology, aiming to optimize the corrosion-resistant and antibacterial properties of intraoral orthodontic materials (Figure 1). The dose-dependent process and the regulation mechanism were investigated and discussed, which laid a considerable foundation for designing efficient fixed appliances and provided guidance for developing advanced dental alloys.

## 2 Materials and methods

### 2.1 Characterization of GO

The morphology and dimension of GO nanosheets (XFNANO Materials Tech Co., China) were detected by SEM (Zeiss Sigma 300, Germany) and AFM (Dimension Icon, Germany), respectively. Raman spectroscopy (Renishaw, United Kingdom) was used to determine the molecular structure of GO at 514 nm wavelength. The functional groups of GO were measured by XPS (Thermo Fisher K-Alpha, United States).

### 2.2 Fabrication and characterization of PDA-GO-coated NiTi alloys

Ni-55.75 wt% NiTi alloys (10 × 10 × 2 mm^3^) and 0.019 × 0.025-inch NiTi archwires with a 13-mm length were obtained from Baoji Titanium Industry (China). The NiTi metals were ground with sandpaper, cleaned with acetone, anhydrous ethanol, and deionized water, and dried for later use. The fresh GO solution (0.2, 0.5, 1.0, 1.5, and 2.0 mg/mL) was ultrasonically dispersed and mixed with an equal volume of 2 mg/mL dopamine hydrochloride in pH 8.5 Tris-HCl buffer to prepare a co-deposition solution. A series of PDA-GO coatings were performed via the self-assembly reaction at 25 °C. After 24 h, the obtained PDA-GO-coated NiTi alloys were rinsed with distilled water and dried at 40 °C. The samples prepared by 0.2, 0.5, and 1.0 mg/mL GO were denoted as PDA-GO1, PDA-GO2, and PDA-GO3, respectively.

The surface morphology and composition of modified NiTi alloys were identified by SEM and EDS. AFM was employed to detect the three-dimensional morphology and roughness. The functional groups were characterized by FTIR. XPS characterized the element content and chemical bonds of PDA-GO-coated NiTi alloys. Mechanical stability and adhesion performance were assessed through the tape-peeling test following the ATSM D3359 standard (Yang et al., 2019). Briefly, the surface form the grid by vertical scratches and was pressed with 3 M adhesive tape, which was examined by optical imaging after the tape was pulled up.

### 2.3 Corrosion resistance performance

#### 2.3.1 Stress corrosion tests

The bending stress of NiTi wire under orthodontic application was simulated using a three-point bending model. The mechanical constant applied to the archwire was 3.0-mm stress when immersed in artificial saliva at 37 °C (Liu et al., 2014). After 4 weeks, the leaching solution was collected, and the release of Ni^2+^ was detected by an inductively coupled plasma mass spectrometry (ICP-MS, Agilent 7700s, United States). After the archwire was unloaded and dried, the corrosion morphology was characterized by SEM, and the surface elements were detected by EDS.

#### 2.3.2 Electrochemical corrosion experiments

During electrochemical tests, different NiTi samples were welded with copper wire and packaged with epoxy resin as the working electrode in a three-electrode system (Metrohm Autolab PGSTAT302N, Swiss). Prior to the potentiodynamic polarization test, the samples were kept in artificial saliva at 37 °C for 0.5 h to obtain a steady state. Tafel polarization curve scanning rate was 1.0 mV/s, voltage range was −1.5 to 1.5 V (vs. Ag/AgCl). The corrosion potential (*E*_*corr*_) and corrosion current density (*I*_*corr*_) were obtained by Tafel extrapolation method.

### 2.4 Antibacterial activity assays

#### 2.4.1 Live/dead staining

*Streptococcus mutans* (*S. mutans*, ATCC25175) were incubated with different NiTi samples for 24 h. According to the Live/Dead bacterial kit (Thermo Fisher Scientific Inc., United States) instructions, each sample was stained with a mixed Brain Heart Infusion (BHI) liquid medium containing STYO9 and PI reagents and incubated for 15 min. The dead bacteria were marked as red, while the live bacteria were marked as green under confocal laser scanning microscopy (CLSM, Leica, Germany).

#### 2.4.2 SEM

After 24 h of co-culture with *S. mutans*, the NiTi samples were washed with PBS and fixed with 2.5% glutaraldehyde for 2 h. After alcohol gradient dehydration and drying, the number and morphology of bacteria on the sample were observed by SEM. Three fields of view images were taken for each substrate.

#### 2.4.3 Colony forming unit (CFU) counting

According to GB/T 2591 standard, 50 μL of bacterial solution (1.0 × 10^5^ CFU/mL) was added to 24-hole plates, with three parallel samples in each group. The surface of the sample was completely immersed, covered with polyethylene film, and incubated in an anaerobic environment for 24 h. The biofilm was dissociated by ultrasonication in PBS for 3 min, and the bacterial solution was diluted in a series of gradients. 50 μL was coated on the BHI solid medium and cultured anaerobically for 24 h. The corresponding CFU values and sterilization efficiency were calculated.

### 2.5 Biocompatibility assessment

Based on ISO10993-12 standard, the extraction DMEM medium was obtained at 3 cm^2^/mL (volume ratio of NiTi surface area to extraction liquid). To evaluate cell proliferation activity, mouse fibroblast L-929 cells were seeded in 96-well plates at a density of 5×10^3^ cells/well and cultured in an incubator at 37 °C for 24 h. The cell lines present in this study were obtained from Zhongqiao Xinzhou Co., Ltd., Shanghai China. 100 μL extract from different samples was added to each well, and DMEM medium was added to the blank control group. After 1, 3, and 5 days of co-culture, the reagents were added according to the instructions of CCK-8 (Dojindo, Japan) and incubated for 1 h. The absorbance at 450 nm was measured. Live/dead staining assays were used to assess the morphology of L-929 cells. Cells were cultured with extract for 5 days and stained with a Calcein-AM/PI Double Stain Kit (Solarbio, China). Then, cells were imaged under an inverted fluorescence microscope (DMI8A, Leica). To evaluate ROS production, L-929 cells were inoculated into 6-well plates at a density of 2×10^5^ cells per well, and extracts of different groups were added. After 24 h of co-culture, a diluted 100 μL DCFH-DA (Sigma-Aldrich, United States) probe was added to each well. After incubation, the fluorescence intensity was detected by flow cytometry (ThermoFisher DxFLEX, United States).

### 2.6 Statistical analysis

Data are represented as mean ± standard deviation, and statistical analyses were realized by GraphPad Prism. Significant differences were identified by one-way analysis of variance (ANOVA) or a two-sided unpaired Student’s t-test. *p* < 0.05 indicated statistical significance.

## 3 Results and discussion

### 3.1 Characterization of GO

**FIGURE 2:**
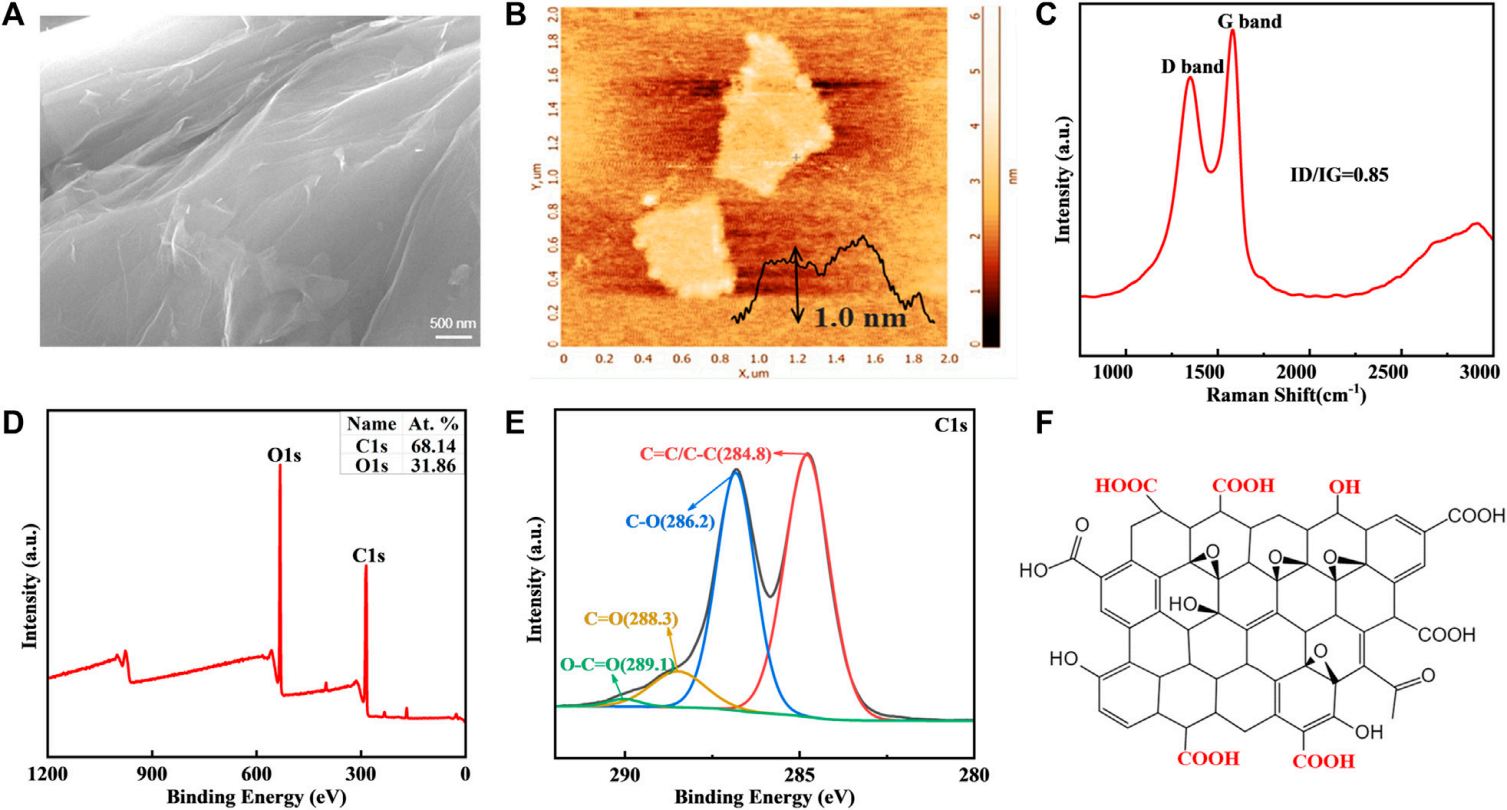
GO characteristics. **(A)** SEM image of GO. **(B)** Representative AFM image. **(C)** Raman spectra. **(D,E)** Elements and functional groups detected by XPS. **(F)** Chemical struture of GO.

Characterization of nanomaterials is crucial for developing biocompatible nanocoatings. SEM reveals that the commercially obtained GO is lamellar with wrinkles on the surface (Figure 2A). The thickness of GO sheet measured by AFM is around 1.0 nm (Figure 2B). According to Raman spectra, GO has two typical peaks, D peak and G peak, which are positioned at 1,350 cm^−1^ and 1,581 cm^−1^, respectively (Figure 2C). The ratio of their intensities is 0.85. The XPS-determined percentage of O element in GO is 31.86%, while the remainder is C element (Figure 2D). GO benefits from various hydrophilic oxygenic groups, including C-O (286.2 eV), C=O (288.3 eV), and O-C=O (289.1 eV), which provide active sites for covalent or non-covalent modification and endow it with high bioavailability (Figures 2E, F) (Hashemi et al., 2018). However, flaky GO nanosheet is easily wrinkled, and direct application will lead to poor adhesion between the coating and metal substrate (Han et al., 2018). The GO-based nanocomposite coating is more adaptable to modified NiTi archwires in orthodontic scenarios.

### 3.2 Coating morphology analysis

Inspired by mussel chemistry, self-polymerized PDA emanates as a versatile modifier with strong adhesion towards substrate surfaces due to the presence of catecholamine. In the self-assembly nanosystems, PDA and GO vividly serve as mortar and brick, respectively. Nanocomposites cannot be stuck on substrate at a low ratio of PDA/GO, while the fixed depositions (‘brick’) are limited at a high ratio (Yu et al., 2023). Based on the biomedical potential of GO, this study aims to explore the medical function of NiTi alloys in oral scenarios obtained by varying GO concentrations.

**FIGURE 3:**
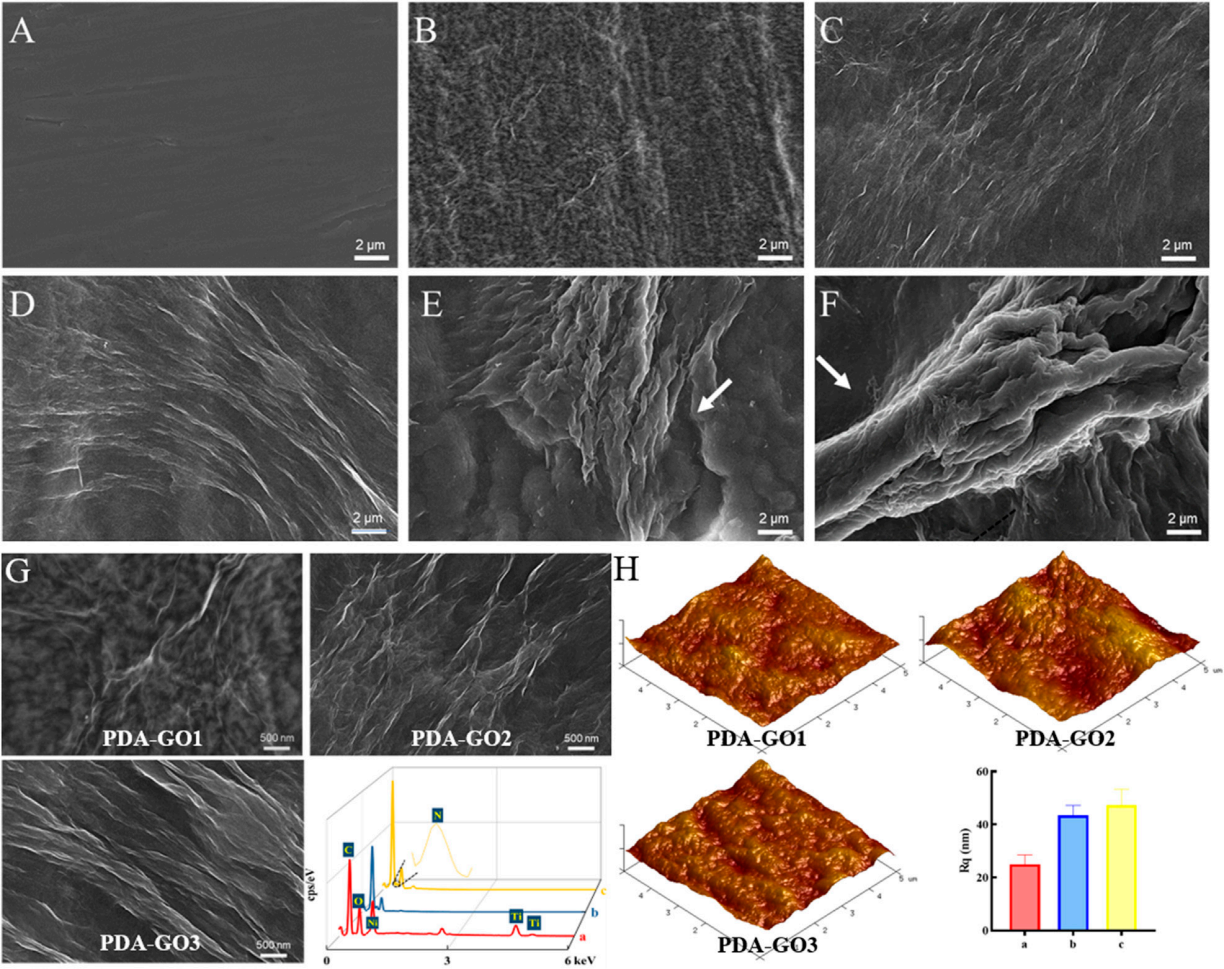
Coating morphology analysis. **(A)** SEM images of uncoated NiTi. **(B–F)** Modified NiTi assembled in DA solution with 0.2–2.0 mg/mL GO. **(G)** Enlarged SEM surface micrographs and EDS spectra of PDA-GO1, PDA-GO2, and PDA-GO3. **(H)** AFM images and roughness detection. Rq: the root mean square roughness.

As shown in SEM images, the surface of bare NiTi sheet possessed some obvious scratches (Figure 3A). The wrinkled deposits were relatively evenly distributed when the GO solution was in the range of 0.2 mg/mL-1.0 mg/mL in the co-deposition system (Figures 3B–D). An increase in GO concentrations (1.5 and 2.0 mg/mL) resulted in agglomerated and stacked aggregates (Figures 3E, F), indicating that the excessive PDA-GO tended to form on the surface protrusions. Some uncovered areas were found in these two groups, which were not conducive to improving the following anti-corrosion performance of NiTi alloys. Therefore, 1.5 and 2.0 mg/mL were not included in the subsequent study.

Based on the SEM and AFM results, scatter deposition covered areas of the PDA-GO1 sample (0.2 mg/mL) with a roughness Rq value of about 24.99 nm (Figures 3G, H). More corrugated deposits in axial directions were observed on the surface of PDA-GO2 (0.5 mg/mL). EDS tests revealed that Ni and Ti element contents decreased compared to that of PDA-GO1, indicating that composite coating was fixed on a wider range of NiTi surface after introducing more GO. For the PDA-GO3 (1.0 mg/mL) sample, AFM displayed a uniformly pleated structure with a slightly increased Rq value, owing to more surface deposition of PDA-GO sheets. In line with our findings, Zhu et al. found that a compact lamellar PDA-GO microstructure was visible under increased GO concentration (Zhu et al., 2017). The different topographies might be attributed to the supplemented defects by appropriate proportion of PDA/GO. During the process, self-assembled PDA adsorbed certain amounts of flexible GO and cross-linked to co-deposit on the substrate (Li et al., 2023). Taken together, morphology analysis demonstrated that a greater GO concentration in a particular range provides a stable solution environment for PDA-GO to diffuse and self-assemble dense and well-arranged layers on NiTi surface.

### 3.3 Chemical structure and adhesive property

**FIGURE 4:**
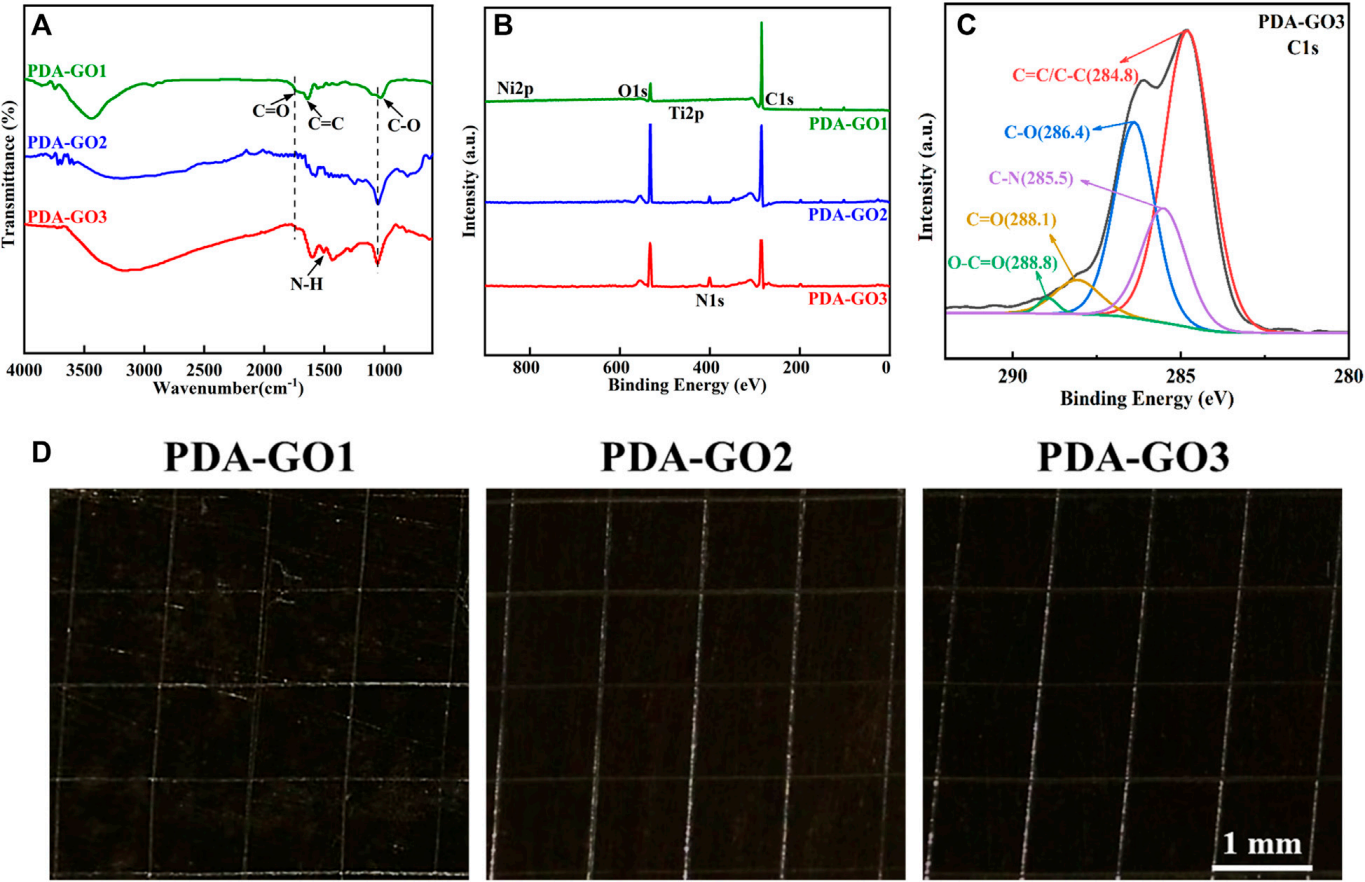
Chemical structure and adhesive property. **(A)** FTIR spectroscopic analysis. **(B)** Element compositions measured by XPS. **(C)** C1s XPS spectra of PDA-GO3. **(D)** Optical microscope images of adhesive tests.

To explore the interaction between PDA and GO, we further analyzed the chemical characteristics of the as-prepared NiTi alloys. FTIR spectra suggested that PDA-GO1 had characteristic peaks at 1,737, 1,623, and 1,029 cm^−1^, corresponding to the stretching vibration peak of C=O, C=C, and C-O, respectively (Figure 4A). The presence of these oxygen-containing functional groups and aromatic rings indicated the GO layers were successfully grafted. With the increase in GO concentration, the characteristic peak of C=O gradually decreased, suggesting that GO was partially reduced by PDA (Han et al., 2022). Compared to that of other groups, the spectrum of PDA-GO3 showed an N-H band at 1,508 cm^−1^, which was derived from the amino group of PDA. The broad peak at 3,100–3,500 cm^−1^ may be caused by -OH (Zhao et al., 2019).

Five element peaks, including C1s, N1s, O1s, Ti2p, and Ni2p, were found on the NiTi-surface-assembled PDA-GO coatings via XPS analysis (Figure 4B). Among them, PDA-GO3 possessed the highest N content, accounting for 6.91%, with an emerged C-N peak at 285.5 eV (Figure 4C). It implied PDA covalently attached to GO on the modified NiTi surface, piling and interpenetrating the layers. Meanwhile, dense distribution of oxygenic groups offered anchoring sites for PDA-GO reaction (Chen et al., 2020). Mechanically, PDA stems from the oxidation, intramolecular cyclization and rearrangement of dopamine, serving as a stabilizer in GO reduction. GO with abundant oxygenic groups could be introduced to the surface by dopamine and may bind to NiTi alloy through π−π stacking and covalent interaction (Jia et al., 2016). Simultaneously, more GO nanosheets as oxidants promoted the nucleation and growth of the PDA film so that the composite coatings completely covered the NiTi alloy, which corresponded well with SEM observations.

Previous coatings, such as alumina, titanium dioxide, and hydroxyapatite, are easily cracked and will be partially peeled off, making them unsuitable for current clinical practice (Araujo et al., 2022). Tape-peeling test was utilized to evaluate the mechanical stability and bonding strength according to ASTM D3359 standard (Shen et al., 2019). As shown in Figure 4D, the PDA-GO1 coating was partially detached from the substrate and the scratch area, and the area percentage was about 12.5%, while the PDA-GO2 and PDA-GO3 coatings displayed no detached or delaminated sediment. The adhesion levels between the latter coatings and the metal substrate were both regarded as 5A, which could be attributed to the compact and aligned structure during the self-assembly process (Figure 4D). This kind of excellent mechanical stability and adhesive performance of coated NiTi lays the foundation for long-term stability while correcting misplaced teeth in orthodontic treatment.

### 3.4 Corrosion resistance performance

#### 3.4.1 Stress corrosion tests

**FIGURE 5:**
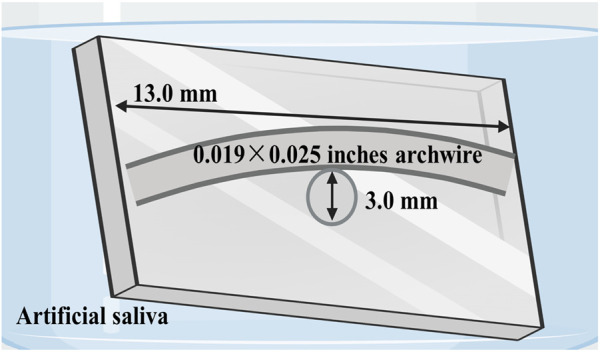
Schematic illustration of bending stress models in artificial saliva designed to simulate intraoral conditions.

**FIGURE 6:**
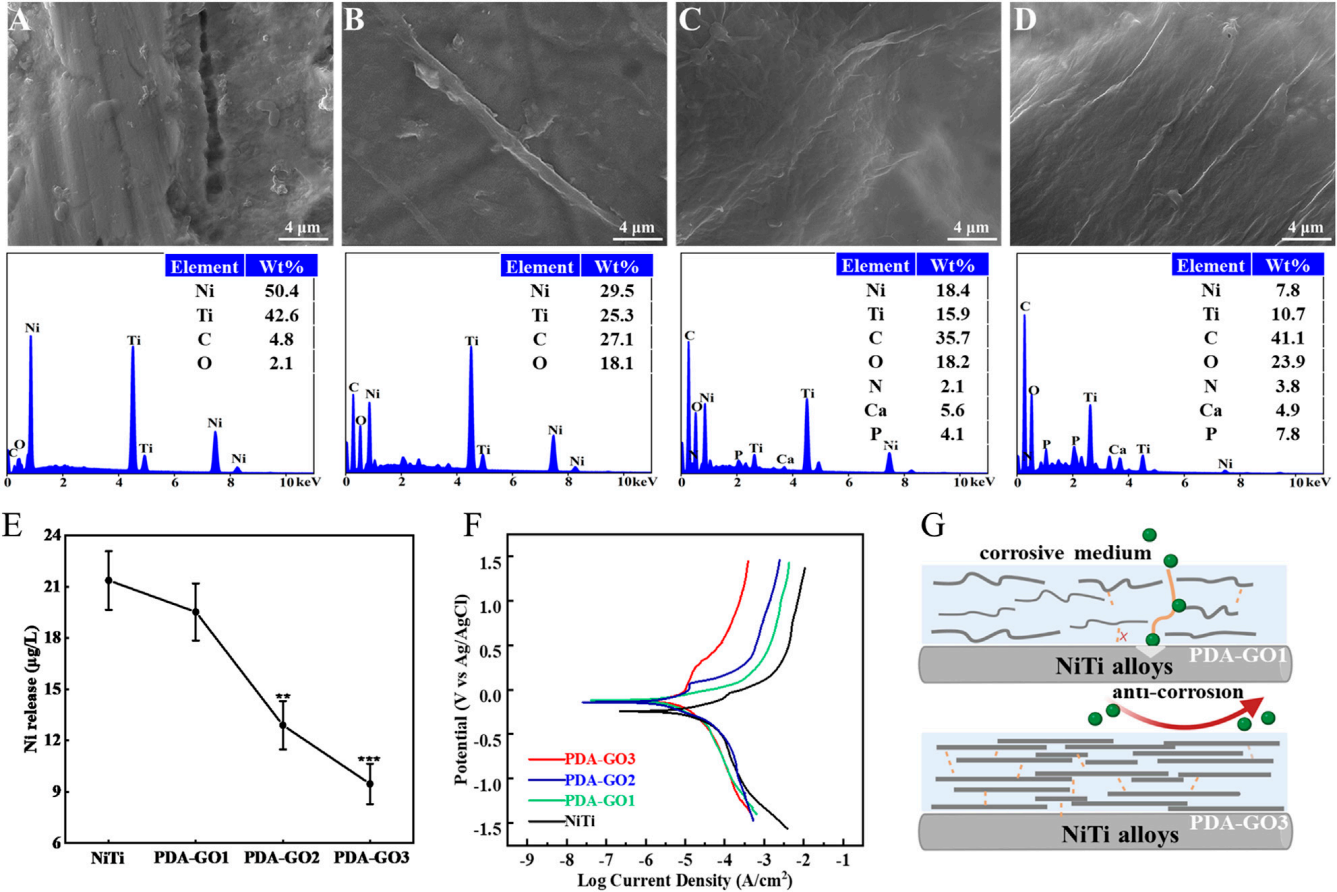
Anti-corrosive properties of PDA-GO coatings. **(A–D)** SEM morphology observations and EDS elemental tests of different substrates, including bare NiTi, PDA-GO1, PDA-GO2, and PDA-GO3, after stress corrosion. **(E)** Corresponding Ni^2+^ concentration. **p < 0.01; ***p < 0.001 compared with NiTi group. **(F)** Representative cyclic potentiodynamic polarization curves of PDA-GO modified NiTi. **(G)** The proposed protection mechanisms during the corrosion process. Certain amounts of GO could chemically and physically bond with PDA and form a labyrinth-like superposition on NiTi surface, significantly reducing the contact area between the orthodontic appliances and corrosion medium.

The oral cavity is a harsh corrosive environment for orthodontic devices, in which mechanical and chemical stresses damage their service life and orthodontic efficacy. To investigate the anti-corrosion potential of PDA-GO coated NiTi, a three-point stress model combined with an artificial saliva corrosion medium was performed, which realistically simulated the service state of archwires in the inoral cavity (Figure 5). SEM showed that corrosion pits and cracks were observed on bare NiTi, suggesting the dissolution of substrate by artificial saliva under 3-mm dislocation for 4 weeks (Figure 6A). Among the modified samples, the PDA-GO1 surface was rough with a relatively high proportion of Ni and Ti elements (Figure 6B). Under the same stress and saliva conditions, the other two samples showed a relatively intact and smooth surface with the granular products composed of calcium and phosphate from saliva, which obtained potent resistance to further development of corrosion (Figures 6C, D). GO possesses ultra-high tensile strength and elastic modulus, and maintains the integrity of its structure even if the archwires undergo stress deformation (Tasnim et al., 2017). Additionally, the binding affinity of PDA not only compensates for the GO defects through bond interactions but also firmly chelates metal ions (Zhu et al., 2022).

After 4 weeks of immersion in artificial saliva, the release of Ni^2+^ in PDA-GO2 and PDA-GO3 samples was 12.90 and 9.47 μg/L, respectively (Figure 6E). Compared with bare NiTi, PDA-GO modified NiTi exhibited remarkable anti-nickel release properties in saliva scenario. Similar results were reported in titanium dioxide-modified NiTi alloys via anodic oxidation technology, but large-scale device manufacture would be difficult (Xu et al., 2019). This research adopted self-assembly to mildly coat PDA-GO, which avoids the oxidation of considerable Ni into the coating and directly reduces Ni^2+^ dissolution. Appropriate GO sheets in PDA-GO3 were wrapped up to effectively form a close lamellar structure for mitigating serious corrosion. Accordingly, interaction of assembly and the well-dispersed morphology make PDA-GO provide stable barrier protection to intraoral stress and salivary environment.

#### 3.4.2 Electrochemical corrosion

Electrochemical corrosion has been considered to be the main reason for Ni^2+^ dissolution in NiTi alloys (Shabalovskaya et al., 2009), which were examined under the saliva medium in the following study. For the polarization curve, the smaller *I*_*corr*_ reflects a slower corrosion rate and good corrosion resistance. As shown in Table 1 and Figure 6F, the *E*_*corr*_ of the unmodified NiTi sample was −171.70 mV, and the *I*_*corr*_ was 3.54 × 10^−6^ A/cm^2^. In marked contrast, *I*_*corr*_ decreased in the PDA-GO coating samples. In particular, the *I*_*corr*_ of PDA-GO3 sample decreased to 5.3 × 10^−7^ A/cm^2^, outperforming other films. The gathered data showed that PDA-GO modification made NiTi alloy have better corrosion resistance in oral corrosion medium. GO sheets were deposited on NiTi by PDA modification under the action of self-assembly, and the saliva electrolyte tended to approach the substrate through unevenness and defects between different layers (Chu et al., 2019). The PDA-GO1 coating had defects with low adhesive properties, which limited improvement in corrosion resistance. Consistent with the stress corrosion results, PDA-GO3 sample possessed the best protective performance in saliva environment, which reflected the influence of PDA/GO parameters on corrosion resistance of NiTi. Specifically, increased GO concentration contributes to orderly accumulating multiple layers during the deposition process, and more GO sheets are crosslinked to seal the NiTi surface by the interlocking effect with PDA, thereby prolonging the diffusion path of corrosive electrolytes (such as chloride ions) into the substrate (Figure 6G) (Zhao et al., 2019). Other factors, such as the interlayer spacing and lattice structure of PDA-GO, might affect corrosion behavior of the coatings, which needs future study (Alkhouzaam et al., 2021).

**TABLE 1:** *E*_*corr*_ and *I*_*corr*_ values calculated from potentiodynamic polarization curves.

Group	*E*_*corr*_ (mV vs. Ag/AgCl)	*I*_*corr*_ (A/cm^2^)
NiTi	−171.70	3.54 × 10^−6^
PDA-GO1	−140.96	2.51 × 10^−6^
PDA-GO2	−109.90	1.05 × 10^−6^
PDA-GO3	−104.77	5.30 × 10^−7^

### 3.5 Antibacterial activity

**FIGURE 7:**
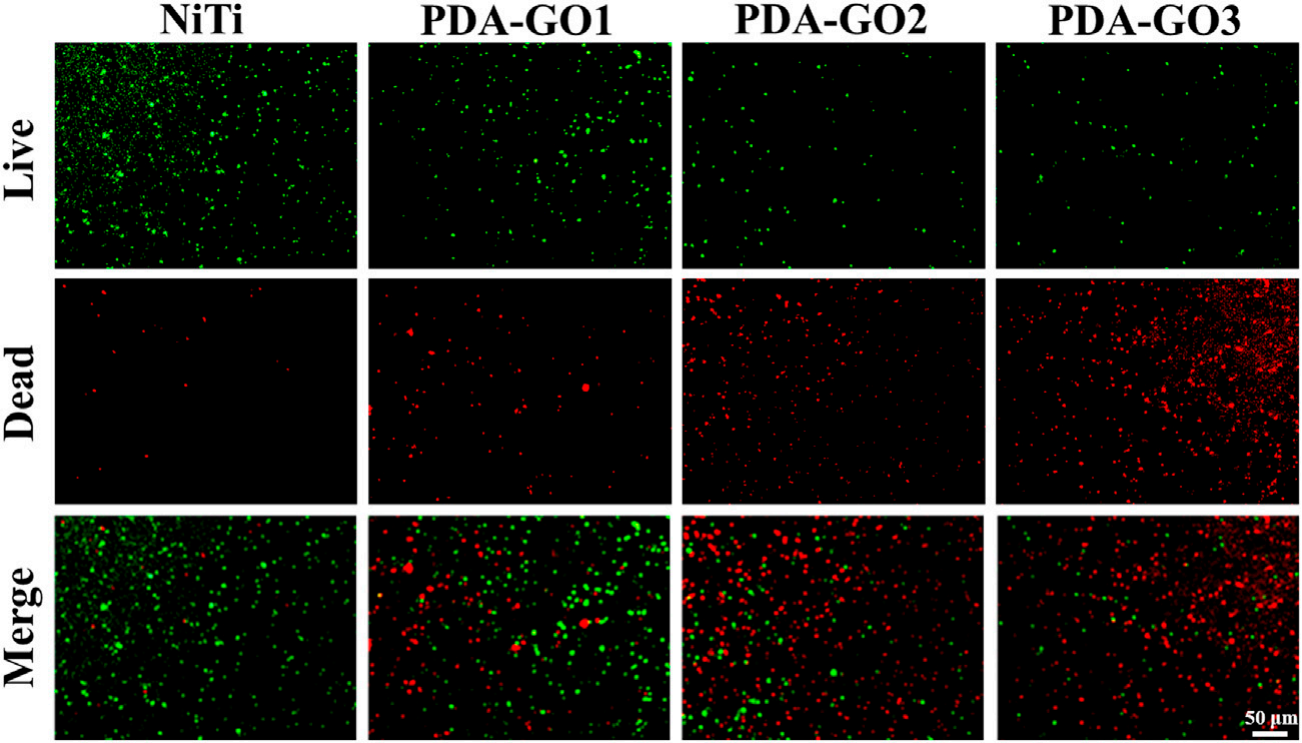
Live/dead results.

Orthodontic brackets and archwires create retention sites for bacterial growth due to their irregular surfaces; thus, proper modification is urgently desired to reduce microbial affection. *S. mutans*, the most critical cariogenic bacteria, was listed as the primary research object of antibacterial testing (Peng et al., 2022). Live/dead bacteria fluorescence staining showed that considerable green-stained live bacteria were observed on the uncoated NiTi surface, with almost no dead bacteria (Figure 7). However, the application of PDA-GO coating decreased the number of live bacteria and increased dead bacteria, exhibiting the property of effectively killing the adhered bacteria after 24 h of contact with modified NiTi.

**FIGURE 8:**
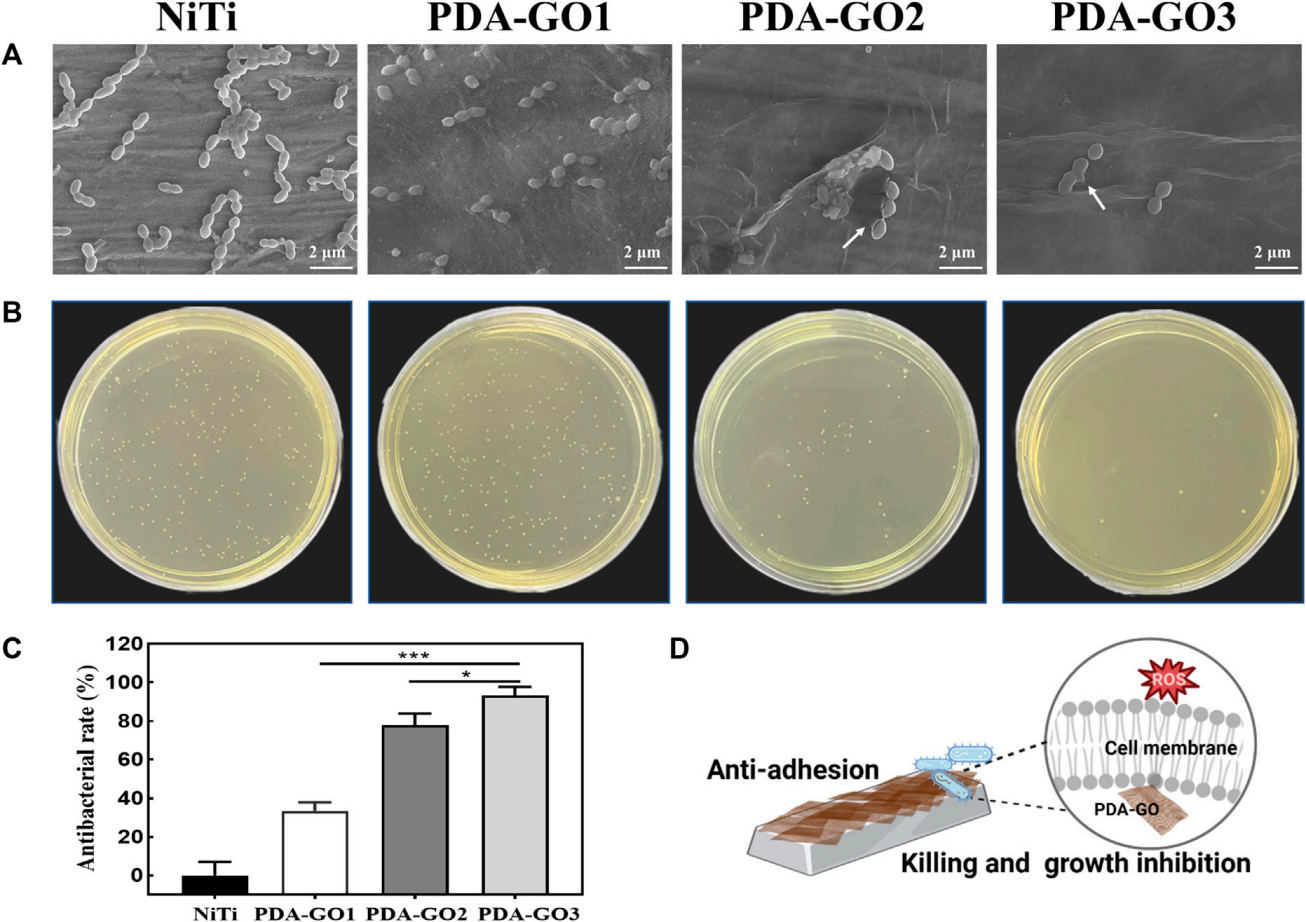
Antibacterial activity assays. **(A)** SEM analysis. **(B)** CFU results and **(C)** relative antibacterial rates. *p < 0.05; ***p < 0.001 compared with NiTi group. **(D)**The anti-adhesive and killing effect of PDA-GO coated NiTi alloys.

SEM images indicated that more *S. mutans* adhered to the surface of bare NiTi, and grew in clusters with a relatively intact surface morphology (Figure 8A). In contrast, adherent bacteria decreased on the PDA-GO1, and no apparent aggregation was observed. PDA-GO2 found sporadic bacteria with changed morphology. On the surface of PDA-GO3, the integrity of *S. mutans* was destroyed at the interface in contact with the coating, as reflected in the exuded cellular content. The results revealed the concentration-dependent bactericidal potential of PDA-GO together with a decreased number of total adherent bacteria. Similarly, the number of bacteria adhered to the surface of bare NiTi was the highest in the CFU experiment (Figure 8B). The number of bacteria in the PDA-GO1 and PDA-GO2 groups gradually decreased, while the bacteria observed corresponding to PDA-GO3 were the least, showing superior antibacterial ability through interfering cell membranes (Zheng et al., 2018). According to the quantitative evaluation (Figure 8C), the antibacterial rates of each PDA-GO coating sample were 33.4%, 77.7%, and 93.1%, respectively, suggesting the tendency for more dead bacteria dependent on GO concentration.

Although efficient antibacterial coatings have been proposed, quite a few present defects, such as complex manufacturing processes or unsatisfied biocompatibility, which seriously hinder clinical applications. As an antibiotic-free antibacterial agent, the mechanism of GO includes two categories: oxidative stress and physical membrane damage such as nanoknife and encapsulation (Gao et al., 2022). It has been proposed that the more GO layers in GO composites, the stronger the oxidative stress in cells (Qiu et al., 2017; Mahmoudi et al., 2019). The increase in GO concentration may induce more PDA-GO layers with higher intracellular ROS levels, producing peroxides that affect the respiratory chain reaction, and destroy the integrity of bacterial membranes. These function cooperatively in the prominent antibacterial performance of PDA-GO3 (Figure 8D). The developed composite nano-coating optimizes the antibacterial properties of orthodontic NiTi alloys by increasing the PDA-GO component, which provides a viable preventive measure against orthodontic complications. Future research may involve elucidating how the PDA-GO alters antibacterial effects at the molecular level.

### 3.6 Biocompatibility properties

**FIGURE 9:**
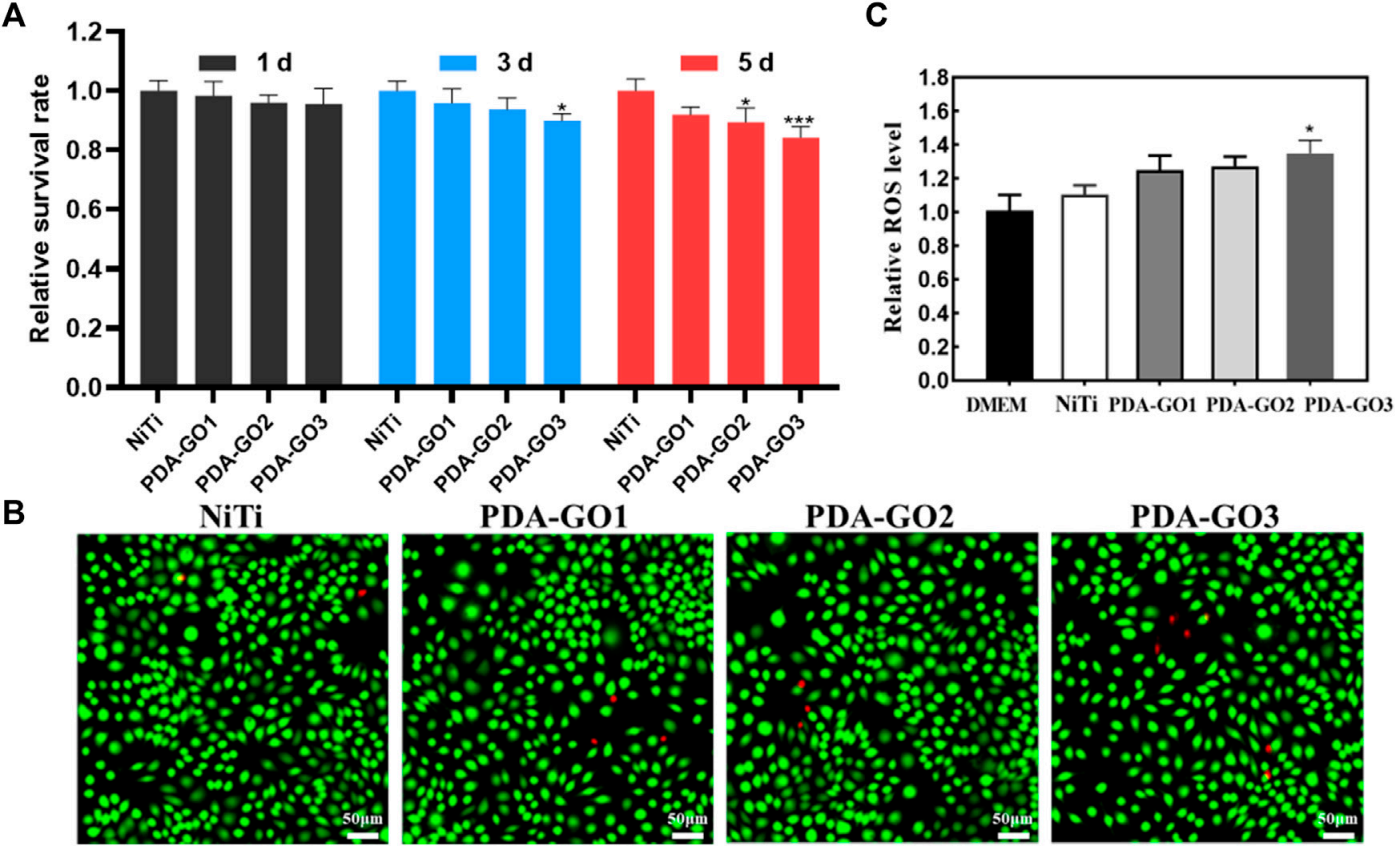
Biocompatibility assessment. **(A)** CCK-8 results. **(B)** Live/dead fluorescence staining after L-929 cells were cultured for 5 days in different NiTi extract. **(C)** Cell ROS production was evaluated and quantified. *p < 0.05; ***p < 0.001 compared with NiTi group.

The biological security of GO composites needs to be highly valued for future clinical translation. To quantitatively determine the cytotoxicity, CCK-8 experiment was performed using L-929 cells, a common ideal *in vitro* model for detecting materials’ biocompatibility (Yu et al., 2017; Nedeljkovic et al., 2022). Based on the results, the relative cell survival rate decreased with the prolongation of culture time. Notably, the cell viability in the PDA-GO3 group at 5 days significantly decreased to about 85% (Figure 9A), which was still acceptable cytotoxicity. The results qualified the modified NiTi as biosafety, with 70% viability being a mark for safe devices (Łyczek et al., 2023). Live/dead staining was carried out to further visualize the cell responses. As shown in Figure 9B, most of the extract-treated L-929 cells were green stained and alive, which was in accordance with the CCK-8 results. Cells in the PDA-GO groups presented a spindle-shaped morphology, similar to cells in the control group. Accordingly, the coatings exhibit not only great bacteriostatic effects, but also appropriate biocompatibility, which may be due to differences in the sensitivity of the cell lines used, the degree of interaction with cells, and the complexity of cellular responses (Zhao et al., 2016). Eukaryotic cells have been shown to be flexible and adaptable to different surfaces, leading to a superior survival rate (Butler et al., 2023). Bacterial membranes possess a higher population of negative intrinsic curvature lipids than mammalian cell membranes and are more likely to be damaged (Zhu et al., 2018). Antibacterial properties correlate positively with a certain range of GO concentration, but the concentration is not as high as possible in terms of biocompatibility. Thus, optimizing the bioefficacy of dental alloys requires a delicate balancing act between the cytotoxicity and bioactivity of different nanocomposite designs (Zheng et al., 2018).

GO-based nanocomposite can induce cytotoxicity through oxidative stress, and the ROS productions of different samples were also judged. PDA-GO coated NiTi stimulated cellular ROS production more than that in bare NiTi, and gradually increased with more GO concentration. From Figure 9C, the intracellular ROS level in PDA-GO3 was about 1.35 times that of the unmodified NiTi group (*p < 0.05). Modified NiTi remained safe for L-929 cells and met ISO 10993-5:2009 requirements for medical devices. This may be related to the fact that the amount of ROS induced is lower than the cell antioxidant threshold (Richtera et al., 2015). In addition, the phenolic hydroxyl and amino groups of PDA can interact with most molecules and proteins, enhancing the materials’ affinity with cells (Liu et al., 2021). PDA-GO coatings applied in NiTi had a high guarantee of safety, but they still lacked clinical trials and long-term follow-up observation.

## 4 Conclusion

This study explores an effective means to optimize orthodontic NiTi archwires by preparing PDA-GO coatings. The morphology, chemical structure, and multifunctional properties are adjusted appropriately by changing the ratio of PDA/GO. PDA-GO is yarn-wrinkled and unevenly distributed at a low GO concentration, which has limited effect on improving properties of NiTi alloys. By appropriately increasing GO concentration, a uniform and dense lamellar structure was obtained. The inherent attributes and special structure of PDA-GO endow the substrate with anti-corrosion property in oral environment and antibacterial capacity against oral cariogenic bacteria. PDA-GO nanocoatings on NiTi alloy combine manufacturing simplicity, adhesive performance, excellent corrosion resistance, reliable antibacterial, and biocompatible properties, which are potential for application as an effective and protective material for orthodontics.

## Data Availability

The raw data supporting the conclusion of this article will be made available by the authors, without undue reservation.

## References

[B1] Alkhouzaam A. Qiblawey H. Khraisheh M. (2021). Polydopamine functionalized graphene oxide as membrane nanofiller: spectral and structural studies. Membranes, 11. 10.3390/membranes11020086 33513669 PMC7910935

[B2] Araujo A. F. Ferreira M. V. F. Felisberto M. D. V. Sicupira D. C. Santos L. A. (2022). Corrosion resistance of a superelastic NiTi alloy coated with graphene–based coatings. Prog. Org. Coatings 165, 106727. 10.1016/j.porgcoat.2022.106727

[B3] Butler J. Handy R. D. Upton M. Besinis A. (2023). Review of Antimicrobial Nanocoatings in Medicine and Dentistry: mechanisms of action, biocompatibility performance, safety, and benefits compared to antibiotics. ACS Nano 17, 7064–7092. 10.1021/acsnano.2c12488 37027838 PMC10134505

[B4] Chen Y. Ren B. Gao S. Cao R. (2020). The sandwich-like structures of polydopamine and 8-hydroxyquinoline coated graphene oxide for excellent corrosion resistance of epoxy coatings. J. colloid interface Sci. 565, 436–448. 10.1016/j.jcis.2020.01.051 31982710

[B5] Cheng W. Zeng X. Chen H. Li Z. Zeng W. Mei L. (2019). Versatile Polydopamine Platforms: synthesis and promising applications for surface modification and advanced nanomedicine. ACS Nano 13, 8537–8565. 10.1021/acsnano.9b04436 31369230

[B6] Chu J. H. Tong L. B. Zhang J. B. Kamado S. Jiang Z. H. Zhang H. J. (2019). Bio-inspired graphene-based coatings on Mg alloy surfaces and their integrations of anti-corrosive/wearable performances. Carbon 141, 154–168. 10.1016/j.carbon.2018.09.047

[B7] Gao Y. Kang K. Luo B. Sun X. Lan F. He J. (2022). Graphene oxide and mineralized collagen-functionalized dental implant abutment with effective soft tissue seal and romotely repeatable photodisinfection. Regen. Biomater. 9, rbac024. 10.1093/rb/rbac024 35529047 PMC9071057

[B8] Ghazal A. R. Hajeer M. Y. Al-Sabbagh R. Alghoraibi I. Aldiry A. (2015). An evaluation of two types of nickel-titanium wires in terms of micromorphology and nickel ions' release following oral environment exposure. Prog. Orthod. 16, 9. 10.1186/s40510-015-0081-1 26061986 PMC4437993

[B9] Han K. Bai Q. Zeng Q. Sun N. Zheng C. Wu W. (2022). A multifunctional mussel-inspired hydrogel with antioxidant, electrical conductivity and photothermal activity loaded with mupirocin for burn healing. Mater. Des. 217, 110598. 10.1016/j.matdes.2022.110598

[B10] Han L. Sun H. Tang P. Li P. Xie C. Wang M. (2018). Mussel-inspired graphene oxide nanosheet-enwrapped Ti scaffolds with drug-encapsulated gelatin microspheres for bone regeneration. Biomater. Sci. 6, 538–549. 10.1039/c7bm01060e 29376156

[B11] Hashemi M. Omidi M. Muralidharan B. Tayebi L. Herpin M. J. Mohagheghi M. A. (2018). Layer-by-layer assembly of graphene oxide on thermosensitive liposomes for photo-chemotherapy. Acta Biomater. 65, 376–392. 10.1016/j.actbio.2017.10.040 29109030

[B12] Jia Z. Shi Y. Xiong P. Zhou W. Cheng Y. Zheng Y. (2016). From Solution to Biointerface: graphene self-assemblies of varying lateral sizes and surface properties for biofilm control and osteodifferentiation. ACS Appl. Mater. Interfaces 8, 17151–17165. 10.1021/acsami.6b05198 27327408

[B13] Krishnan V. Ravikumar K. K. Sukumaran K. Kumar K. J. (2012). *In vitro* evaluation of physical vapor deposition coated beta titanium orthodontic archwires. Angle Orthod. 82, 22–29. 10.2319/040811-251.1 21749248 PMC8881040

[B14] Lee H. A. Ma Y. Zhou F. Hong S. Lee H. (2019). Material-independent surface chemistry beyond polydopamine coating. Accounts Chem. Res. 52, 704–713. 10.1021/acs.accounts.8b00583 30835432

[B15] Lee J. H. Jo J. K. Kim D. A. Patel K. D. Kim H. W. Lee H. H. (2018). Nano-graphene oxide incorporated into PMMA resin to prevent microbial adhesion. Dent. Mater 34, e63–e72. 10.1016/j.dental.2018.01.019 29402540

[B16] Li X. Wu C. Hou B. Wu J. Sun R. Chen M. (2023). Molecular investigation of interplay mechanism between polydopamine and graphene oxide: the effect of oxidation degree on the adsorption behavior of polydopamine. Appl. Surf. Sci. 611, 155759. 10.1016/j.apsusc.2022.155759

[B17] Liu J.-K. Liu I. H. Liu C. Chang C.-J. Kung K.-C. Liu Y.-T. (2014). Effect of titanium nitride/titanium coatings on the stress corrosion of nickel–titanium orthodontic archwires in artificial saliva. Appl. Surf. Sci. 317, 974–981. 10.1016/j.apsusc.2014.08.132

[B18] Liu X. Chen W. Shao B. Zhang X. Wang Y. Zhang S. (2021). Mussel patterned with 4D biodegrading elastomer durably recruits regenerative macrophages to promote regeneration of craniofacial bone. Biomaterials 276, 120998. 10.1016/j.biomaterials.2021.120998 34237507

[B19] Łyczek J. Bończak B. Krzymińska I. Giżyński K. Paczesny J. (2023). Gold–oxoborate nanocomposite‐coated orthodontic brackets gain antibacterial properties while remaining safe for eukaryotic cells. J. Biomed. Mater. Res. Part B Appl. Biomaterials 111, 996–1004. 10.1002/jbm.b.35208 36462180

[B20] Mahmoudi E. Ang W. L. Ng C. Y. Ng L. Y. Mohammad A. W. Benamor A. (2019). Distinguishing characteristics and usability of graphene oxide based on different sources of graphite feedstock. J. colloid interface Sci. 542, 429–440. 10.1016/j.jcis.2019.02.023 30771638

[B21] Mazinani A. Nine M. J. Chiesa R. Candiani G. Tarsini P. Tung T. T. (2021). Graphene oxide (GO) decorated on multi-structured porous titania fabricated by plasma electrolytic oxidation (PEO) for enhanced antibacterial performance. Mater. Des. 200, 109443. 10.1016/j.matdes.2020.109443

[B22] Močnik P. Kosec T. Kovač J. Bizjak M. (2017). The effect of pH, fluoride and tribocorrosion on the surface properties of dental archwires. Mater Sci. Eng. C Mater Biol. Appl. 78, 682–689. 10.1016/j.msec.2017.04.050 28576038

[B23] Moses J. C. Mandal B. B. (2022). Mesoporous silk-bioactive glass nanocomposites as drug eluting multifunctional conformal coatings for improving osseointegration and bactericidal properties of metal implants. ACS Appl. Mater Interfaces 14, 14961–14980. 10.1021/acsami.2c00093 35320670

[B24] Nedeljkovic I. Doulabi B. Z. Abdelaziz M. Feilzer A. J. Exterkate R. A. M. Szafert S. (2022). Cytotoxicity and anti-biofilm properties of novel hybrid-glass-based caries infiltrant. Dent. Mater 38, 2052–2061. 10.1016/j.dental.2022.11.018 36437129

[B25] Peng S. Sang T. Wang H. Guan Y. Deng Y. Wang P. (2022). Bioinspired anti-demineralization enamel coating for orthodontics. J. Dent. Res. 101, 1620–1627. 10.1177/00220345221129806 36271659

[B26] Qiu J. Geng H. Wang D. Qian S. Zhu H. Qiao Y. (2017). Layer-number dependent antibacterial and osteogenic behaviors of graphene oxide electrophoretic deposited on titanium. ACS Appl. Mater Interfaces 9, 12253–12263. 10.1021/acsami.7b00314 28345852

[B27] Richtera L. Chudobova D. Cihalova K. Kremplova M. Milosavljevic V. Kopel P. (2015). The composites of graphene oxide with metal or semimetal nanoparticles and their effect on pathogenic microorganisms. Materials 8, 2994–3011. 10.3390/ma8062994

[B28] Shabalovskaya S. A. Tian H. Anderegg J. W. Schryvers D. U. Carroll W. U. Van Humbeeck J. (2009). The influence of surface oxides on the distribution and release of nickel from Nitinol wires. Biomaterials 30, 468–477. 10.1016/j.biomaterials.2008.10.014 18996586

[B29] Shen Y. Wu Y. Tao J. Zhu C. Chen H. Wu Z. (2019). Spraying Fabrication of Durable and Transparent Coatings for Anti-Icing Application: dynamic water repellency, icing delay, and ice adhesion. ACS Appl. Mater Interfaces 11, 3590–3598. 10.1021/acsami.8b19225 30589262

[B30] Sun A. R. Sun Q. Wang Y. Hu L. Wu Y. Ma F. (2023). Surface modifications of titanium dental implants with strontium eucommia ulmoides to enhance osseointegration and suppress inflammation. Biomaterials Res. 27, 21. 10.1186/s40824-023-00361-2 PMC10022180 36927570

[B31] Tanaka H. Dotera T. Hyde S. T. (2023). Programmable self-assembly of nanoplates into bicontinuous nanostructures. ACS Nano 17, 15371–15378. 10.1021/acsnano.2c11929 37527198 PMC10448885

[B32] Tasnim N. Kumar A. Joddar B. (2017). Attenuation of the *in vitro* neurotoxicity of 316L SS by graphene oxide surface coating. Mater Sci. Eng. C Mater Biol. Appl. 73, 788–797. 10.1016/j.msec.2016.12.123 28183673 PMC5312756

[B33] Venkatesan K. Kailasam V. Padmanabhan S. (2020). Evaluation of titanium dioxide coating on surface roughness of nickel-titanium archwires and its influence on Streptococcus mutans adhesion and enamel mineralization: a prospective clinical study. Am. J. Orthod. Dentofac. Orthop. 158, 199–208. 10.1016/j.ajodo.2019.07.019 32576426

[B34] Wang C. Zhang G. Li Z. Xu Y. Zeng X. Zhao S. (2019). Microtribological properties of Ti6Al4V alloy treated with self-assembled dopamine and graphene oxide coatings. Tribol. Int. 137, 46–58. 10.1016/j.triboint.2019.04.030

[B35] Wang N. Yu J. Yan J. Hua F. (2023). Recent advances in antibacterial coatings for orthodontic appliances. Front. Bioeng. Biotechnol. 11, 1093926. 10.3389/fbioe.2023.1093926 36815889 PMC9931068

[B36] Xu J. L. Lai T. Luo J. M. (2019). Preparation and characterization of the aesthetic coating on nickel-titanium orthodontic archwire by electrophoretic deposition. Prog. Org. Coatings 137, 105271. 10.1016/j.porgcoat.2019.105271

[B37] Yang N. Yang T. Wang W. Chen H. Li W. (2019). Polydopamine modified polyaniline-graphene oxide composite for enhancement of corrosion resistance. J. Hazard. Mater. 377, 142–151. 10.1016/j.jhazmat.2019.05.063 31158583

[B38] Yang P. Yu F. Yang Z. Zhang X. Ma J. (2022). Graphene oxide modified κ-carrageenan/sodium alginate double-network hydrogel for effective adsorption of antibiotics in a batch and fixed-bed column system. Sci. total Environ. 837, 155662. 10.1016/j.scitotenv.2022.155662 35525355

[B39] Yu P. Lei J. Honglei L. Hui C. (2017). Cell death affected by dental alloys: modes and mechanisms. Dent. Mater. J. 36, 82–87. 10.4012/dmj.2016-154 27928106

[B40] Yu Y. Zhao X. Ye L. (2023). A novel biocompatible wearable sensors based on poly (vinyl alcohol)/graphene oxide hydrogel with superior self-adhesion, flexibility and sensitivity. Compos. Struct. 309, 116768. 10.1016/j.compstruct.2023.116768

[B41] Zhang R. Han B. Liu X. (2023). Functional Surface Coatings on Orthodontic Appliances: reviews of friction reduction, antibacterial properties, and corrosion resistance. Int. J. Mol. Sci. 24, 6919. 10.3390/ijms24086919 37108082 PMC10138808

[B42] Zhao C. Pandit S. Fu Y. Mijakovic I. Jesorka A. Liu J. (2016). Graphene oxide based coatings on nitinol for biomedical implant applications: effectively promote mammalian cell growth but kill bacteria. RSC Adv. 6, 38124–38134. 10.1039/c6ra06026a

[B43] Zhao Z. Guo L. Feng L. Lu H. Xu Y. Wang J. (2019). Polydopamine functionalized graphene oxide nanocomposites reinforced the corrosion protection and adhesion properties of waterborne polyurethane coatings. Eur. Polym. J. 120, 109249. 10.1016/j.eurpolymj.2019.109249

[B44] Zheng H. Ma R. Gao M. Tian X. Li Y. Q. Zeng L. (2018). Antibacterial applications of graphene oxides: structure-activity relationships, molecular initiating events and biosafety. Sci. Bull. 63, 133–142. 10.1016/j.scib.2017.12.012 36658925

[B45] Zhou Z. Seif A. Pourhashem S. Silvestrelli P. L. Ambrosetti A. Mirzaee M. (2022). Experimental and theoretical studies toward superior anti-corrosive nanocomposite coatings of aminosilane wrapped layer-by-layer graphene Oxide@MXene/waterborne epoxy. ACS Appl. Mater Interfaces 14, 51275–51290. 10.1021/acsami.2c14145 36321761

[B46] Zhu J. Yuan L. Guan Q. Liang G. Gu A. (2017). A novel strategy of fabricating high performance UV-resistant aramid fibers with simultaneously improved surface activity, thermal and mechanical properties through building polydopamine and graphene oxide bi-layer coatings. Chem. Eng. J. 310, 134–147. 10.1016/j.cej.2016.10.099

[B47] Zhu M. Liu P. Shi H. Tian Y. Ju X. Jiang S. (2018). Balancing antimicrobial activity with biological safety: bifunctional chitosan derivative for the repair of wounds with Gram-positive bacterial infections. J. Mater Chem. B 6, 3884–3893. 10.1039/c8tb00620b 32254316

[B48] Zhu Z. Zhao Y. Liu Y. Wu C. Lei Y. Su W. (2022). Improvement of anticorrosion properties of waterborne coatings by designing a new type of modified graphene with high dispersion and low conductivity. Prog. Org. Coatings 173, 107188. 10.1016/j.porgcoat.2022.107188

